# Prevalence of low vitamin D levels in patients with Hidradenitis suppurativa in Jordan: A comparative cross-sectional study

**DOI:** 10.1371/journal.pone.0265672

**Published:** 2022-03-18

**Authors:** Khaled Seetan, Batool Eldos, Muthanna Saraireh, Rami Omari, Yousef Rubbai, Anas Jayyusi, Maha Abu Jubran

**Affiliations:** 1 Department of Clinical Sciences, Faculty of Medicine, Yarmouk University, Irbid, Jordan; 2 Princess Aisha Bint Al-Hussein College of Nursing and Health Sciences, Al-Hussein Bin Talal University, Maan, Jordan; Clinic for Infectious and tropical diseases, Clinical centre of Serbia, SERBIA

## Abstract

Hidradenitis suppurativa (HS) is a chronic inflammatory disease of the apocrine gland bearing skin, presenting various stages of flexural skin pain, erythema, painful nodules, abscesses, sinuses, and fistulas. We aimed to assess serum vitamin D levels in patients with (HS) in Jordan. a cross-sectional comparative study conducted among 110 patients with HS and 110 matched controls, who didn’t previously receive vitamin D therapy. Serum vitamin D was measured and classified into normal (>30 ng/ml), insufficient (20–30 ng/ml), and low (<20 ng/ml). The mean age of the cases was 43.1 ± 12.9 years and the mean disease duration was 19.4 months. The mean body mass index among patients with HS was 30 and about 34% of them were smokers. The mean Vitamin D level was 8.4 ng/ml and all HS patients were vitamin D deficient. Patients of HS were more likely to have vitamin D deficiency compared to healthy controls. Most of the study subjects and particularly all of the patients with HS have low vitamin D levels. Smoking and high BMI, were associated with HS. We suggest the implementation standard public dietary recommendations of Vitamin D supplementation, smoking cessation, and weight reduction behaviors with further assessment of disease course among HS patients.

## Introduction

Vitamin D is a fat-soluble steroid hormone essential for human health [1]. Not only just it has an established role in mineral balance and skeletal maintenance [2]. But also, a protective factor in several diseases like hypertension, diabetes, cardiovascular diseases, autoimmune diseases, and cancers [2]. Furthermore, Vitamin D plays multiple skin functions ranging from keratinocyte proliferation, differentiation, and apoptosis to barrier maintenance and immune-regulatory processes [3], making its supplementation useful in the management of various dermatologic conditions, including HS [4].

Low vitamin D level has been classified into two categories. According to the US endocrine society guidelines, vitamin D deficiency is considered as serum level of less than 20ng/ml (50 nmol/L), while vitamin D level between 21-29ng/ml (52.5–72.5nmol/L) is vitamin D insufficiency [5]. In general, low sun exposure is considered as major risk factor for Vitamin D deficiency along with female gender and high BMI, and decreased physical activities. While genetic polymorphism has also been reported [6–9].

In recent years, there has been a growing interest in the role played by vitamin D in skin disease [10]. Imbalance of vitamin D level has been associated with skin disorders like including atopic dermatitis, psoriasis, vitiligo, polymorphous light eruption, mycosis fungoides, alopecia areata, systemic lupus erythematosus, melanoma, and Hidradenitis suppurativa (HS) [11], but the evidence was deemed speculative and inconclusive [12]. Moreover, despite the lack of literature, it was reported that vitamin D level was found to be lower in HS patients compared to the other population.

Hidradenitis suppurativa (HS) "also known as acne inversa," is a chronic, recurrent, and debilitating inflammatory skin condition [13], characterized by recurrent inflammatory nodules and abscesses due to follicular structural abnormalities that augmented by risk factors such as smoking, obesity, positive family history, and shaving [14]. Lesions usually affect apocrine gland-bearing anatomical areas of the body and is characterized by painful, inflamed nodules or boils that progress to abscesses, sinus tracts, and scarring [15, 16]. HS has a psychologically and clinically significant adverse effect on the patient’s quality of life [17].

In general, there is a scarcity of evidence assessing the level of vitamin D and its association with HS. Similarly, there is a lack of relevant literature in Jordan. Thus, this study aimed to assess serum vitamin D levels in patients with Hidradenitis suppurativa in Jordan.

## Materials and methods

A cross-sectional comparative study was conducted among patients of the Department of Dermatology, Jerash Hospital, between April 2018 and March 2020. The study included 110 patients diagnosed with HS and 110 healthy individuals who did not receive any vitamin D supplements over the last year were recruited and included in the study. The healthy individuals, with almost the same age (+ 3 years) and sex as with the matched cases, served as the controls.

The data collected using structured questionnaire including sociodemographic characteristics in form of age, gender, smoking status, and concurrent chronic illness; HS duration was also recorded. Serum 25-hydroxyvitamin D3 (25(OH)D) concentration was assessed by electrochemiluminescence binding assay using the Elecsys Vitamin D total II Cobas by Roche. Per the vitamin D level, the status of vitamin D was classified into the following categories: normal (>30 ng/mL), insufficient (20–30 ng/mL), and low (<20 ng/mL) [3, 5, 18] using the reference values at the lab where samples were analyzed.

Data were collected, organized, and statistical analysis was performed using the statistical package for social sciences (SPSS) version 25. Continuous variables were represented as means and standard deviations, while categorical variables were reported as frequencies and percentages. Chi-square tests were used to compare categorical variables (gender, smoking, and vitamin D status) between patients with HS and controls. Additionally, an independent-samples t-test was used to compare continuous variables (age, BMI, and vitamin D level) between cases and controls. Logistic regression analysis was used to determine the effect of vitamin D level, age, gender, BMI, and smoking status on disease state. A P-value of less than 0.05 was considered statistically significant for all purposes.

This study was conducted ethically in accordance with the World Medical Association Declaration of Helsinki. Participation in the study was voluntary, and patients were consented to participate in the study after explaining research purposes and procedures and informed that refusal to participate would not affect the care provided to them. Written informed consent was obtained from all participants to participate in the study. The research protocol was reviewed and approved by the institutional research body–King Abdullah University Hospital- Jordan with an ID 13-1-2285.

## Results

A total of 220 participants were recruited for the study (110 cases and 110 controls) with mean age of 43.1 ± 12.9 years and 40.2 ± 8.9 years for cases and control respectively. More than half of the study participants were females (54.5%). The mean BMI was 30 and 22.2 for the case and control groups, respectively. The mean duration of the disease among the case group was 19.4 ± 11 months. Nearly third (34.5%) of cases group were smokers. Most of the study participants have had no chronic diseases (78.2%), while those with chronic medical and dermatological conditions representing 15.9%, and 5.9%, respectively (Table 1).

**Table 1:** Demographic and clinical data of study participants.

		Study group									
		Case			Control			Total			*p* (*X*^*2*^*/t*)
		N	N %	M (SD)	N	N %	M (SD)	N	N %	M (SD)	
Gender	Male	50	45.5%		50	45.5%		100	45.5%		1.0 (0.00)
	Female	60	54.5%		60	54.5%		120	54.5%		
Smoking status	Non-smoker	34	30.9%		65	59.1%		99	45.0%		< 0.001* (17.65)
	Smoker	76	69.1%		45	40.9%		121	55.0%		
Comorbid conditions	Chronic medical condition	14	12.7%		21	19.1%		35	15.9%		0.397 (1.8)
	Chronic dermatological condition	6	5.5%		7	6.4%		13	5.9%		
	None	90	81.8%		82	74.5%		172	78.2%		
BMI				30.0 (4.6)			22.2 (2.6)			26.1 (5.4)	< 0.001* (15.5)
BMI classification	Underweight	0	0.0%		12	10.9%		12	5.5%		< 0.001* (100)
	Healthy weight	20	18.2%		72	65.5%		92	41.8%		
	Overweight	32	29.1%		26	23.6%		58	26.4%		
	Obesity	58	52.7%		0	0.0%		58	26.4%		
Vitamin D level				8.4 (3.0)			17.6 (10.4)			13 (8.9)	< 0.001* (8.9)
Vitamin D level classification	Low	110	100.0%		72	65.5%		182	82.7%		< 0.001* (45.9)
	Insufficient	0	0.0%		22	20.0%		22	10.0%		
	Normal	0	0.0%		16	14.5%		16	7.3%		

**Fig 1:**
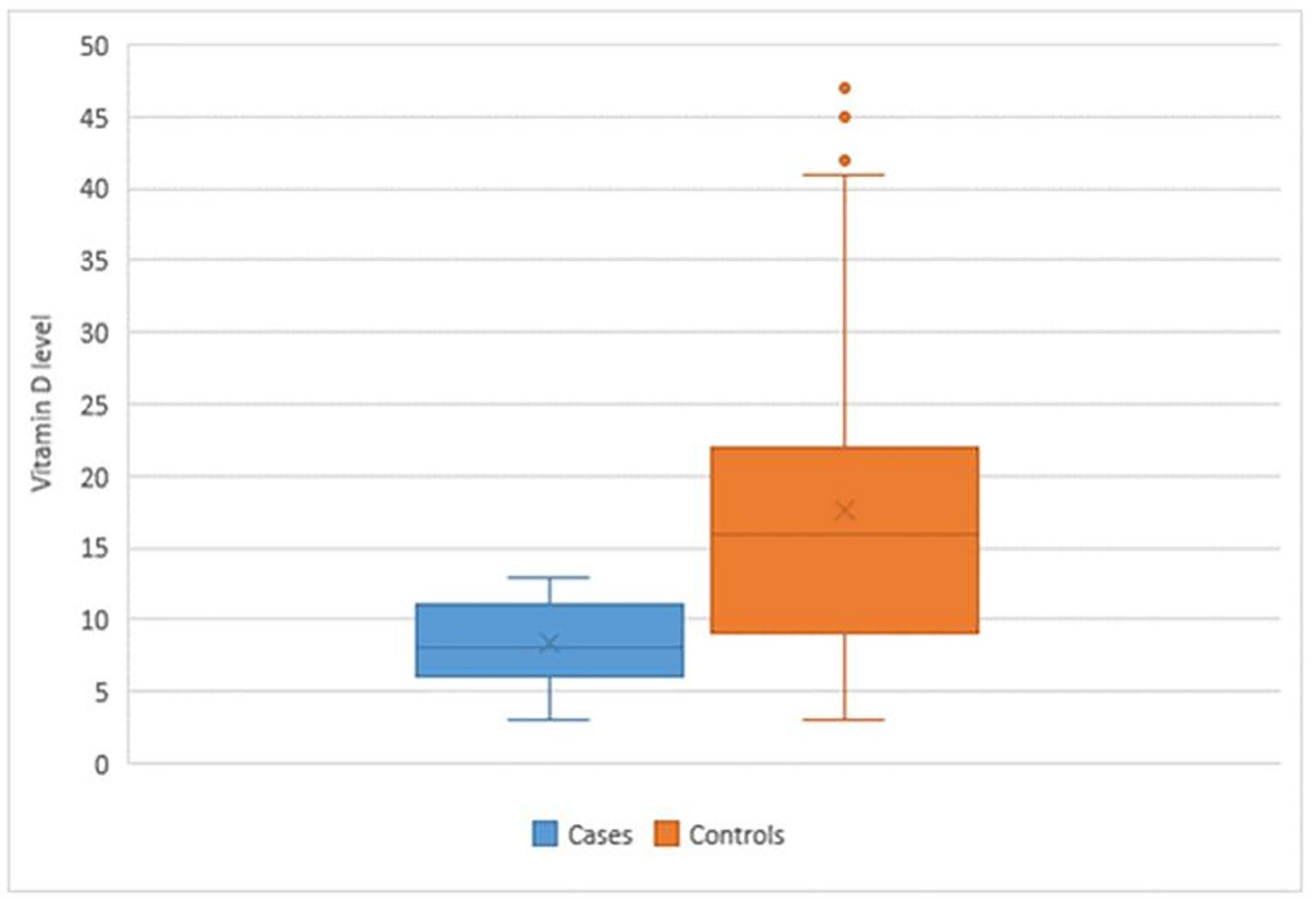
Boxplot showing distribution of vitamin D level between cases and controls.

The mean vitamin D level among the control group was 17.6 ng/ml, while the mean in the case group was 8.4 ng/ml. Regarding Vitamin D level in the Control group, about 16 (14.5%) of them have had an average level of vitamin D. All of the participants in the case group were found to have a low level of vitamin D, compared to 72 individuals (65.5%) of the control group (Table 1; Fig 1). Statistical Analysis showed that individuals with average higher BMI were more likely to suffer from HS disease than individuals with lower average BMI (p-value <0.001). Similarly, participants who were smokers were more likely to have the disease compared to non-smoker (p-value <0.001), while cases with HS disease were more likely to suffer vitamin D deficiency compared to the control group (p-value < 0.001). No significant correlation was noted between vitamin D level and disease severity (r = 0.08, p = 0.406) (Table 1).

Linear logistic regression analysis showed significant negative correlation between vitamin D level and smoking (beta = -0.183, p = 0.004), and BMI (beta = -0.322, p < 0.001) after adjusting for age and gender (Table 2).

**Table 2:** Linear regression analysis showing effects of high BMI, and smoking on vitamin D level.

Model		Unstandardized Coefficients		Standardized Coefficients	t	Sig.	95.0% Confidence Interval for B	
		B	Std. Error	Beta			Lower Bound	Upper Bound
1	(Constant)	11.329	2.907		3.897	.000	5.599	17.058
	Age	.076	.054	.094	1.395	.164	-.031	.182
	Gender	-.971	1.209	-.054	-.803	.423	-3.354	1.412
2	(Constant)	28.863	3.986		7.241	.000	21.006	36.720
	Age	.060	.050	.075	1.197	.233	-.039	.159
	Gender	-1.706	1.122	-.095	-1.521	.130	-3.917	.505
	Smoking status	-3.281	1.134	-.183	-2.894	.004	-5.516	-1.047
	BMI	-.534	.105	-.322	-5.082	.000	-.741	-.327

a. Dependent Variable: Vitamin D level

## Discussion

Hidradenitis suppurativa (HS) is a low-prevalent disease characterized by chronic relapsing inflammation. It arises in areas that contain apocrine glands, including the axilla, groin, gluteal, and mammary area [19, 20]. The prevalence of the disease is highly variable among different geographical distributions ranging from 0.03–4%. Locally, data on disease prevalence is not available, but a recent study demonstrated a prevalence of 0.01–2.2% in the Asia-pacific region [21].

Hidradenitis suppurativa is a multifactorial disease with an inflammatory background due to the occlusion of follicles. Genetics and hormonal factors have been reported in the etiology of the disease [22, 23]. In a study done by Brandao et al., it is suggested that malfunction of vitamin D metabolism may play a role in the pathogenesis of HS [24].

Our results showed that all HS patients had vitamin D deficiency, consistent with other studies. In the study of Guillet et al., introducing vitamin supplements have resulted in a significant clinical improvement among patients with HS with vitamin D deficiency. Vitamin D supplementation was also found to decrease the severity of other dermatologic conditions like psoriasis and atopic dermatitis [25]. Slightly less severe deficiency rates were reported by Fabbrocini et al., where 77.5% of his cases were suffering from low levels, and 12.5% had insufficient levels [15].

Although vitamin D deficiency is linked to HS here, this finding may be due to other factors that may alter the serum level along with the disease; hence possible confounding effect and the reported level of vitamin D in Jordan already mentioned. In addition to HS, the effect of vitamin D deficiency and supplementation has been studied in inflammatory skin diseases. It has been shown that vitamin D deficiency is more frequent in psoriasis and atopic dermatitis and that vitamin D supplementation decreases disease severity [26]. The reported decline in vitamin D levels among patients with different dermatological diseases could be explained by decreased synthesis due to the absence of adequate sun exposure resulting from patients’ behavior and attitude such as avoiding undressing in public or being alone in closed places in more deficiency [25]. This can be applicable in our study as sun exposure among female study participants can be influenced by wearing Hijab which is a prevalent practice in Jordan.

Vitamin D level is affected by many factors, including age, ethnicity, climate changes, skin pigmentation, and the presence of concurrent medical conditions. In Jordan, a study done to assess vitamin D levels among the general population reported that the overall prevalence of low vitamin D levels was 89.7% (cut off level = 25(OH)D<30ng/ml), and the deficiency was more prevalent in females [27]. The cultural and religious aspects can explain the higher rates of deficiency among females as Jordanian females wear Hijab and cover their bodies, which may interfere with vitamin D synthesis.

A higher prevalence of smoking was found among the female population, which was markedly high and might have contributed to the development of the disease as the study demonstrated a statistically significant association between smoking and the disease’s development (P-value < 0.001). Smoking was the strongest positive predictor of HS in our study (aOR = 4.197, 95% CI: 1.395–12.628), consistent with other studies [28]. Furthermore, smokers usually present with more severe HS than non-smokers [20, 29]. This may be due to nicotine’s ability to promote mast cell degranulation, neutrophil chemotaxis, and epidermal hyperplasia [30].

BMI was also a significant positive predictor of HS (OR = 2.236, 95% CI: 1.65–3.032). Correlating with multiple studies, most HS patients have truncal and peripheral obesity [31–33]. Our study found a statistically significant association between the disease and BMI. Patients with HS had a high average BMI (P-value <0.001). It is now claimed that the risk of getting HS increases by 1.12 with each rise in BMI [20]. The effect of obesity may result from the mechanical friction of skin folds and subsequent follicular hyperkeratosis, microbial overgrowth, and systemic inflammatory status [34].

As smoking and obesity might play a role in HS pathogenesis, smoking must be discouraged, and a healthier lifestyle must be promoted. This will provide a benefit to the patients with chronic diseases as well as HS patients.

### Limitations of the study

This is an observational study with a small number of patients, and there are possible confounding factors that may alter Serum 25 (OH) -D concentrations including individual genetic variations, pre-existing medical conditions, medication use, dietary habits, and amount of sun exposure in the studied population. Furthermore, although patients with Hidradenitis suppurativa are more likely to be vitamin D deficient, it is difficult to correlate the disease activity with serum vitamin D levels, so randomized controlled trials are needed to assess the effect of vitamin D supplements on patient’s disease behavior.

## Supporting information

S1 Data (XLSX)
